# Subphenotypes of Cardiac Arrest Patients Admitted to Intensive Care Unit: a latent profile analysis of a large critical care database

**DOI:** 10.1038/s41598-019-50178-0

**Published:** 2019-09-20

**Authors:** Zhongheng Zhang, Min Yao, Kwok M. Ho, Yucai Hong

**Affiliations:** 1 Department of Emergency Medicine, Sir Run Run Shaw Hospital, Zhejiang University School of Medicine, Hangzhou, 310016 China; 2 Department of Surgery, Wound Care Clinical Research Program, boston University School of Medicine and Boston Medical Center, Boston, MA 02118 USA; 3 Department of Intensive Care Medicine, Royal Perth Hospital, School of Population & Global Health, University of Western Australia, Perth, WA 6000 Australia

**Keywords:** Cardiovascular diseases, Risk factors

## Abstract

Cardiac arrest (CA) may occur due to a variety of causes with heterogeneity in their clinical presentation and outcomes. This study aimed to identify clinical patterns or subphenotypes of CA patients admitted to the intensive care unit (ICU). The clinical and laboratory data of CA patients in a large electronic healthcare database were analyzed by latent profile analysis (LPA) to identify whether subphenotypes existed. Multivariable Logistic regression was used to assess whether mortality outcome was different between subphenotypes. A total of 1,352 CA patients fulfilled the eligibility criteria were included. The LPA identified three distinct subphenotypes: *Profile 1* (13%) was characterized by evidence of significant neurological injury (low GCS). *Profile 2* (15%) was characterized by multiple organ dysfunction with evidence of coagulopathy (prolonged aPTT and INR, decreased platelet count), hepatic injury (high bilirubin), circulatory shock (low mean blood pressure and elevated serum lactate); *Profile 3* was the largest proportion (72%) of all CA patients without substantial derangement in major organ function. *Profile 2* was associated with a significantly higher risk of death (OR: 2.09; 95% CI: 1.30 to 3.38) whilst the mortality rates of *Profiles 3* was not significantly different from *Profile 1* in multivariable model. LPA using routinely collected clinical data could identify three distinct subphenotypes of CA; those with multiple organ failure were associated with a significantly higher risk of mortality than other subphenotypes. LPA profiling may help researchers to identify the most appropriate subphenotypes of CA patients for testing effectiveness of a new intervention in a clinical trial.

## Introduction

Cardiac arrest (CA) is associated with substantial morbidity and mortality, and most will require intensive care unit (ICU) admission for post-resuscitation care. Evidence suggests that mortality after CA requiring ICU admission remains unacceptably high (50–60%)^1,2^; although for those who survive to hospital discharge, a good neurological outcome is common^3,4^. Despite advances in post-resuscitation care, the improvement in survival outcome after CA has been relatively small^5^. Therapeutic interventions after CA including therapeutic hypothermia, inhaled Xenon, and neuromuscular blockade have been trialed without much success despite promising results in the earlier animal studies^6–9^. One of the many possible reasons for these CA trials to confirm the benefits of these interventions could be due to the problem of selecting the most likely patients who would respond to such interventions. Given the high mortality rate of CA patients, it is possible that most interventions would be deemed futile for the sickest group of patients even when such interventions may offer some benefits to the less severely ill CA patients.

The concept of precision medicine is to customize healthcare, with medical decisions, treatments, practices, or products being tailored to the individual patients. The concept has been well explored in oncology that outcomes have been improved by individualized the chemotherapeutic treatment based on genomic testing^10^. Given ICU patients are usually heterogeneous in many ways, it is conceivable that individualized treatment may improve their outcomes. Currently many critically ill patients are admitted to the ICU with a board diagnostic syndrome such as sepsis, acute respiratory distress syndrome (ARDS) and acute kidney injury with heterogeneous underlying causes, clinical characteristics, outcomes and also possibly response to treatment. Identifying the subphenotypes of ICU patients within a certain diagnostic group may represent a small step towards precision medicine. Recently, some sophisticated machine learning methods, such as latent profile/class analysis, have been employed to identify subphenotypes of ARDS, and suggested that these subphenotypes have different baseline clinical characteristics and response to fluid strategies^11,12^. Similarly, CA patients also exhibit significant heterogeneity, and identification of subphenotypes may help to stratify patients who are most likely to benefit from potential therapies in a clinical trial.

To the best of our knowledge, no studies has been published about how we should classify CA patients into different subphenotypes and whether these subphenotypes are associated with different outcomes. We hypothesized that using latent profile analysis of routinely collected clinical data, we can identify subphenotypes of CA patients that are associated with different clinical outcomes. In this large database cohort study, we aimed to assess how many CA subphenotypes might exist, and describe how their clinical characteristics and outcomes were different.

## Materials and Methods

### Setting

A large US-based critical care database named Medical Information Mart for Intensive Care (MIMIC-III) was used for this study. The description of MIMIC-III is available elsewhere^13^. Briefly, the MIMIC-III database integrated de-identified, comprehensive clinical data of the patients admitted to the ICUs of Beth Israel Deaconess Medical Center in Boston, Massachusetts, from June 1st, 2001 to October 31st, 2012. There were 53,423 distinct hospital admissions for adult patients (aged 16 years or above) admitted to the ICUs during the study period. Since this study was an analysis of the third party anonymized publicly available database with pre-existing institutional review board (IRB) approval, further IRB approval from our institution was exempted. This study was reported according to the REporting of studies Conducted using Observational Routinely-collected health Data (RECORD) Statement^14^.

### Participants

ICU admissions with the diagnosis of cardiac arrest (ICD-9 code: 427.5) were identified for potential eligibility. For a patient with multiple ICU admissions, only the first admission was included in the analysis (e.g. each subject had a unique patient ID, by which duplicated cases could be excluded). Exclusion criteria included: (1) patients younger than 18 years old; (2) hospital stay longer than 200 days; and (3) elective admissions.

### Demographical and laboratory variables

The following variables were extracted from the MIMIC- III database for the first day of ICU admission: age at the time of hospital admission, gender, admission type, ethnicity, type of ICU, urine output, Sequential Organ Failure Assessment (SOFA) score, use of vasopressors (including epinephrine, norepinephrine, dopamine and dobutamine), the lowest Glasgow coma score (GCS) and use of renal replacement therapy (RRT). SOFA score used in this study was calculated within the first 24 hours after ICU admission. If a variable was measured more than once in the first 24 hours, the value associated with the greatest severity of illness was used. For example, the lowest value of mean blood pressure (BP) and GCS reported in the first 24 hours were used in the study.

**Figure 1:**
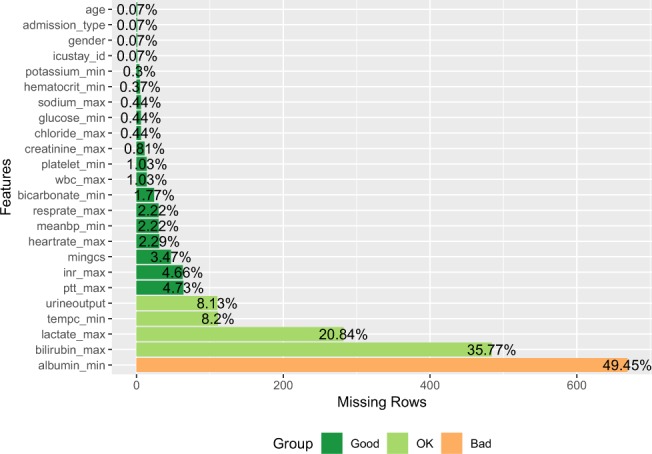
The percentage of missing values for each variable used for latent profile analysis.

Laboratory variables such as lactate, activated partial thrombin time (aPTT), international normalized ratio (INR), sodium, potassium, creatinine, total bilirubin, platelet, hematocrit and bicarbonate were recorded for the first 24 hours after ICU admission. If there were multiple records of a test, the one associated with the greatest severity of illness was obtained. Vitals signs including mean BP, heart rate, respiratory rate, and body temperature were also extracted. The primary outcome of interest was hospital mortality, defined by the survival status of patients at hospital discharge. Secondary outcomes included length of stay (LOS) in ICU and hospital. Missing variables were common in the MIMIC-III database (Fig. 1), and multiple imputations were performed to replace any missing data. We prespecified that variables with more than 50% missing values were excluded from modeling LPA. Multiple imputation was performed by the following steps^15,16^:

1. Fit the data with appropriate model. The variables to be imputed were used as response variable and other relevant variables were used as predictors. We used predictive mean matching for continuous variables and Classification and regression trees for categorical variables.
2. Estimate missing data point using the fitted model in step 1.
3. Repeat the steps 1 and 2 for 5 times for each missing data point.

### Latent profile analysis

Latent profile analysis (LPA) is a kind of unsupervised machine learning technique that aims to recover hidden groups or patterns from observed data. It is similar to clustering techniques but is more flexible. Specifically, LPA is based on explicit modeling of the underlying data, allowing for the identified subgroups to be uncertain^17^. In our study, the observed data were laboratory tests and vital signs of CA patients recorded during the first 24 hours of ICU admission and the hidden groups were latent subphenotypes of CA. Variables included for LPA modeling is primarily based on domain knowledge and literature review, as well as the availability in the database. The key to successful LPA modelling was to choose the number of profiles. In this study, the number of profiles were determined by Bayesian information criteria (BIC), entropy and bootstrap likelihood ratio tests. Specifically, BIC was used to compare the models with different numbers of profiles and/or specifying different parameterizations. Lower values of the BIC are indicative of a better model fit^18^. Entropy ranges from 0 to 1 with a higher value indicates higher classification utility. The Vuong-Lo-Mendell-Rubin Likelihood ratio test (LRT) was used to assess the number of mixture components in a specific finite mixture model parameterization, and p values were reported for the comparison of n-class model with (n-1)-class model^19^. A p-value of 0.05 was used to judge the statistical significance for the likelihood ratio test. Furthermore, because the number of patients should be sizable in each latent class, we pre-specified that the patient proportion in each subphenotype should not be less than 5% in any of the other latent classes^20^. The clinical interpretation was also considered when determining the number of latent classes.

### Statistical analysis

Continuous variables were expressed as the mean (standard deviation) or median (interquartile range) as appropriate, and were compared between the different subphenotypes of CA using analysis of variance (ANOVA)^21^. The *CBCgrps* package was employed for the statistical description and bivariate inference^22^. Clinical outcomes such as the mortality, length of stay (LOS) in ICU and hospital were compared between latent subphenotypes.

Multivariable logistic regression was used to assess whether mortality outcome was different between different subphenotypes after adjusting for important covariates, including the SOFA score, age, ethnicity, type of ICU, mean BP and time era of the patients’ admission (patients admitted from 2008–2012 versus those enrolled before 2008). All statistical analyses were performed using R package (version 3.4.3) and Mplus (version 7.4). A p-value less than 0.05 was considered to be statistically significant.

### Ethics approval and consent to participate

This study was an analysis of the third party anonymized publicly available database with pre-existing institutional review board (IRB) approval.

## Results

### Patient selection

**Figure 2:**
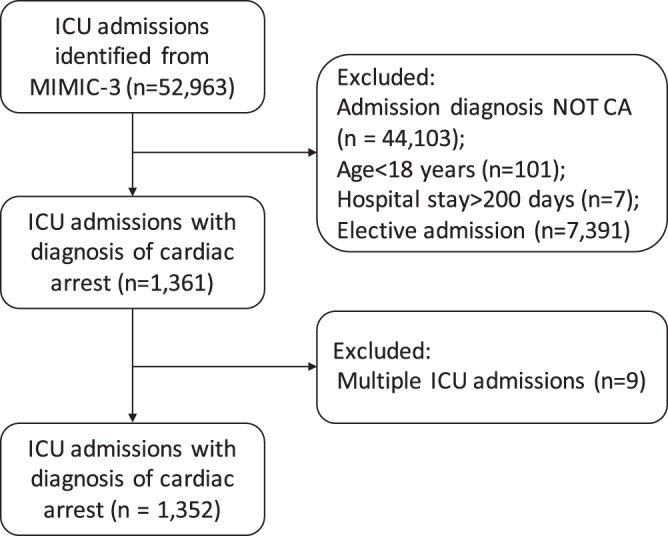
Flow chart of patient selection

We initially identified 52,963 ICU admissions from the MIMIC-III database. After application of exclusion criteria and removal of multiple ICU admissions, a total of 1,352 CA patients were included for analysis (Fig. 2).

### Choose the best number of latent profiles

LPA models with different number of profiles were compared for their fit. The BIC and AIC values decreased rapidly from a 2-profile model to the 3-profile model (dropped by 2000) with only marginal gain when further increase the number of profiles from 4 to 5 (dropped by 1000). However, the entropy remained relatively stable beginning from 3-profile model. When the number of profiles increased to 4 or more, the number of patients in some subphenotypes was less than 5% than in other subphenotypes. The likelihood ratio test showed that a 4-profile model was not significantly better than the 3-profile model. Taken all profile selection criteria together, the 3-profile model was considered as the best model (Table 1).

**Table 1:** Choose the best number of profiles for day 1.

Number of profiles	LL	AIC	BIC	aBIC	Entropy	AICC	P*	Number of patients in each profile (%)						
								1	2	3	4	5	6	7
2	−96714.16	193562.3	193911.3	193698.5	0.902	193569.4	0.2230	241 (18)	1111 (82)					
3	−95836.17	191852.3	192321.2	192035.3	0.945	191865.3	0.0140	175 (13)	208 (15)	969 (72)				
4	−95366.65	190959.3	191548.0	191189.0	0.954	190980.1	0.4395	25 (2)	1100 (81)	220 (16)	7 (1)			
5	−94777.82	189827.6	190536.1	190104.1	0.959	189858.3	0.7600	172 (13)	76 (6)	905 (67)	190 (14)	8 (1)		
6	−94433.30	189184.6	190012.9	189507.8	0.960	189227.3	0.0125	1 (0)	209 (15)	1025 (76)	7 (1)	85 (6)	25 (2)	
7	−93791.13	187946.3	188894.4	188316.2	0.968	188003.3	0.1260	164 (12)	22 (2)	933 (69)	154 (11)	71 (5)	7 (1)	1 (0)

*P value was reported comparing k-profile model to (k-1)-profile model based on the VUONG-LO-MENDELL-RUBIN likelihood ratio test.

Abbreviations: AIC: Akaike Information Criterion; AICC: Akaike Information Criterion corrected; BIC: Bayesian information criteria; aBIC: adjusted Bayesian information criteria.

### Clinical characteristics of the subphenotypes of CA

**Figure 3:**
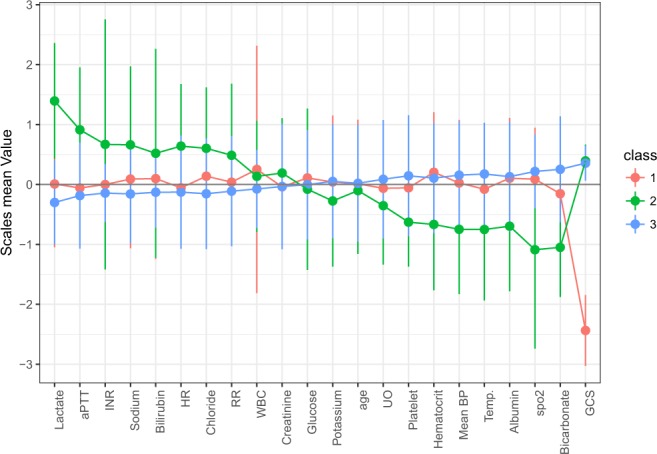
Clinical characteristics of the four latent profiles. Z-score was displayed in the y-axis, which was the value centered by the population mean and scaled by standard deviation. The use of Z-score facilitated the comparisons between variables measured at different scales. Abbreviations: aPTT: activated partial thrombin time; INR: international normalized ratio; HR: heart rate; RR: respiratory rate; WBC: white blood cell count; UO: urine output; BP: blood pressure; GCS: Glasgow Coma Scale; SpO_2_: oxygen saturation of pulse oximetry.

*Profile 3* had the largest proportion of all CA patients (72%) and was considered as the baseline subphenotype to compare with other subphenotypes. *Profile 2* (15%) was characterized by multiple organ dysfunction with evidence of coagulopathy (with prolonged aPTT and INR, decreased platelet count), hepatic injury (with a high bilirubin), renal failure (low urine output and high creatinine) and circulatory shock (with low mean BP and elevated serum lactate). *Profile 1* (13%) was characterized by neurological injury (with a low GCS). Figure 3 shows the Z-score (i.e. centered at population mean and scaled by its standard deviation) of each observed variable, stratified by the latent profiles.

### Clinical outcomes of the subphenotypes of CA

The hospital mortality rate for the whole cohort was 54%. *Profile 2* had the highest hospital mortality rate (76%), followed by *Profile 1* (63%) and *Profile 3* (48%). The baseline subphenotype (*Profile 3*) had the lowest mortality rate (48%). *Profile 3* had the longest length of stay in both ICU (median: 4; IQR: 2 to 8 days) and hospital (median: 9; IQR: 4 to 17 days). There was no significant difference between profiles in age and gender. Patients from *Profile 2* were less likely to be admitted to the coronary care unit (CCU), and *Profile 3* was more likely to be admitted to the CCU. Because *Profile 2* patients showed a high incidence of circulatory shock, the use of vasoactive agents were, as expected, more prevalent compared to the other three profiles (Table 2).

**Table 2:** Baseline characteristics and outcomes by profiles on day 1.

Variables	Total (n = 1352)	Profile 1 (n = 175)	Profile 2 (n = 208)	Profile 3 (n = 969)	p
Age, years (IQR)	69 (58,80)	69 (55,82)	69 (56,79)	69 (59,80)	0.466
Gender, Male (%)	816 (60)	112 (64)	119 (57)	585 (60)	0.400
Ethnicity, n (%)					0.047
ASIAN	34 (3)	9 (5)	8 (4)	17 (2)	
BLACK	114 (8)	14 (8)	24 (12)	76 (8)	
HISPANIC	40 (3)	9 (5)	4 (2)	27 (3)	
UNKNOWN	220 (16)	27 (15)	36 (17)	157 (16)	
WHITE	944 (70)	116 (66)	136 (65)	692 (71)	
Admission period, n (%)					<0.001
Before 2008	763 (56)	69 (39)	119 (57)	575 (59)	
2008 to 2012	589 (44)	106 (61)	89 (42)	394 (41)	
GCS, median (IQR)	15 (14,15)	3 (3,7)	15 (15,15)	15 (15,15)	<0.001
SOFA, median (IQR)	6 (3,9)	10 (7,12)	10 (8,12)	5 (3,7)	<0.001
Mean MBP, median (IQR)	76 (69,83)	78 (70,87)	72 (66,79)	76 (69,83)	<0.001
Minimum MBP, median (IQR)	54 (44,62)	53 (42,62)	42.17 (28,53)	56 (48,63)	<0.001
Type of care unit, n (%)					<0.001
CCU	422 (31)	55 (31)	34 (16)	333 (34)	
CSRU	219 (16)	21 (12)	44 (21)	154 (16)	
MICU	440 (33)	68 (39)	77 (37)	295 (30)	
SICU	143 (11)	18 (10)	27 (13)	98 (10)	
TSICU	128 (9)	13 (7)	26 (12)	89 (9)	
Use of vasoactive agents					
Dopamine, n (%)	272 (20)	33 (19)	75 (36)	164 (17)	<0.001
Epinephrine, n (%)	128 (9)	13 (7)	63 (30)	52 (5)	<0.001
Norepinephrine, n (%)	442 (33)	67 (38)	147 (71)	228 (24)	<0.001
Dobutamine, n (%)	51 (4)	2 (1)	15 (7)	34 (4)	0.006
Clinical outcomes					
Hospital LOS, days (IQR)	8 (3,17)	7 (2,18)	4 (1,11)	9 (4,17)	<0.001
ICU LOS, days (IQR)	4 (2,8)	4 (1,10)	2 (1,7)	4 (2,8)	<0.001
Hospital mortality, n (%)	732 (54)	110 (63)	159 (76)	463 (48)	<0.001

Abbreviations: ICU: intensive care unit; LOS: length of stay; UO: urine output; GCS: Glasgow coma scale; BP: blood pressure. SOFA: sequential organ failure assessment; CCU: coronary care unit; CSRU: cardiac surgery recovery unit; MICU: medical ICU; SICU: surgical ICU; TSICU: Trauma-Surgical ICU; MBP: mean arterial blood pressure.

Multivariable Logistic regression showed that hospital mortality was significantly different between the three latent profiles (Table 3). As compared to the *Profile 1* subphenotyope, *Profile 2* was significantly associated with a higher risk of death (OR: 2.09, 95% CI: 1.30 to 3.38). *Profiles 3* were not significantly different from *Profile 1*. In addition to the LPA profiles, each 10-year increment in age was associated with a 4% increase in risk of death (OR: 1.04, 95% CI: 1.02 to 1.06; p = 0.001), and each point of SOFA score increment was associated with an 8% increase in risk of death (OR: 1.08; 95% CI: 1.04 to 1.12; p < 0.001). Admission period and ethnicity were not significantly associated with mortality.

**Table 3:** Multivariable logistic regression model for profile on day 1.

Variables	OR	Lower limit of 95% CI	Upper limit of 95% CI	P value
Age, with each 10-year increase	1.04	1.02	1.06	0.001
Ethnicity (Asia as reference)				
BLACK	1.17	0.49	2.75	0.723
HISPANIC	0.55	0.20	1.53	0.258
UNKNOWN	1.65	0.72	3.71	0.231
WHITE	1.15	0.52	2.48	0.726
SOFA (with 1-point increase)	1.08	1.04	1.12	0.000
Mean MBP (with each 20-mmHg increase)	0.86	0.70	1.06	0.159
Profile 1 as reference				
Profile 2	2.09	1.30	3.38	0.002
Profile 3	0.79	0.54	1.17	0.239
Admission period (before 2008 as reference)	0.98	0.77	1.24	0.868
Care unit type (CCU as reference)				
CSRU	0.55	0.38	0.78	0.001
MICU	2.22	1.66	2.97	0.000
SICU	1.40	0.94	2.11	0.099
TSICU	2.08	1.36	3.22	0.001

Abbreviations: SOFA: sequential organ failure assessment; CCU: coronary care unit; CSRU: cardiac surgery recovery unit; MICU: medical ICU; SICU: surgical ICU; TSICU: Trauma-Surgical ICU; MBP: mean arterial blood pressure.

## Discussion

Using routinely collected clinical data in a large electronic database, this study could identify three subphenotypes of CA patients. The three subphenotypes were: *Profile 1* (13%) characterized by neurological injury with a low GCS with the first 24 hours of ICU admission; *Profile 2* (15%) characterized by multiple organ dysfunction; *Profile 3* (72%) was characterized by a lowest mortality and considered as the baseline subphenotype. More importantly, the mortality rates were also different between LPA profiles, especially between *Profile 3* (48%) and *Profile 2* (76%). These results have some clinical relevance and require further discussion.

An important feature of Profile 2 was circulatory shock manifested by low BP, elevated lactate, decreased bicarbonate and metabolic acidosis. Recent study showed that metabolic acidosis after cardiac arrest was frequently caused by refractory shock and was associated with a high mortality^23^. The post-cardiac arrest syndrome comprises of four important components including anoxic brain injury, post cardiac arrest myocardial dysfunction, systemic ischemia/reperfusion response, and persistent underlying precipitating pathology^24,25^. Our results suggest that these four components do not affect all CA patients equally. For example, the *Profile 1* in our study was characterized by predominantly isolated neurological injury, while *Profile 2* might be characterized by systemic ischemia/reperfusion response involving multiple organs. Renal dysfunction is an important component in profile 2. It is reported that approximately one in three CA patients may develop acute kidney injury, which has not been consistently found to be associated with mortality outcome^26–28^. The enrollment period appeared to be different among the latent profiles. While profile 3 patients were more likely to be recruited before 2008, profile 1 patients were more likely to be enrolled after 2008 (p < 0.001). Probably, profile 1 patients were those with severe neurological injury and could not survive to ICU admission in older days. With the development of organ supportive techniques such as extracorporeal membrane oxygenation (ECMO), more CA patients can survive the acute phase of cardiac arrest and being treated in ICU.

Our study showed that the clinical patterns, characteristics and outcomes of CA were heterogeneous. Hence, it would be prudent to enroll CA patients into any clinical trials on CA patients based on their LPA profile to maximize the power of the trials. For example, it will be preferable to enroll only patients fitting into *Profile 1* for intervention trials aiming at improving neurological outcomes (e.g. therapeutic hypothermia), and only patients fitting into *Profile 2* for testing interventions aiming at improving circulatory perfusion and renal outcomes. Enrolling patients fitting into *Profile 2* into trials that test interventions to improve neurological outcomes would potentially reduce the power of the studies, with a higher risk of having false negative results. The analysis notes that patients in profile 3 had the longest length of stay, which is attributable to the fact that patients in the other profiles had higher mortality and thus the length of stay was shorter.

This study has some strengths and weaknesses. This large electronic healthcare database study utilized a large number of clinical variables to separate the CA patients by LPA which has not been done before. The obvious disadvantages are inherent to the nature of a retrospective design. First, the missing data was a problem in the database. Using only cases with complete data in all variables would reduce the sample size substantially. We used multiple imputations to preserve power while accounting for the uncertainty induced by the imputation process^15^. Second, only routinely collected clinical variables were included in the LPA model. Some important clinical information such as ischemic time before restoration of spontaneous circulation, and novel neurological biomarkers including neuron-specific enolase, S100b, liver-specific miR-122-5p and myelin basic protein have not been analyzed in the current study^29,30^. Because these biomarkers are not routinely used in clinical practice, and their utility to improve any future LPA modelling of CA patients remains uncertain. For a classification system to be adopted for clinical trial purposes, it is important to use clinical and laboratory variables that are routinely collected in most healthcare institutions. Third, our results do not provide any link between any interventions and its effectiveness on mortality outcome. For example, we observed that *Profile 2* was associated with the highest mortality rate. Whether any interventions can improve the outcomes of this group of patients remains uncertain, but this merits further assessment by adequately-powered randomized controlled trials. Finally, the current study did not allow to distinguish between intra-hospital cardiac arrest (IHCA) and out-of-hospital cardiac arrest (OHCA). It has been observed that OHCA and IHCA can be quite different in clinical presentations and outcomes^31,32^.

## Conclusion

In summary, LPA using routinely collected clinical data could identify three distinct subphenotypes or clinical patterns of CA; those with multiple organ failure were associated with a significantly higher risk of mortality than the baseline subphenotype or neurological injury alone. LPA profiling may help researchers to identify the most appropriate subphenotypes of CA patients for testing effectiveness of a new intervention in a clinical trial.

## Data Availability

Data were fully available in the MIMIC website.
